# Dynamic Bearing Characteristics of Cement Concrete Pavement Under Heavy-Duty Loads

**DOI:** 10.3390/ma19071437

**Published:** 2026-04-03

**Authors:** Wentao Qu, Siyuan Li, Bang Xu, Xiao Huang, Qiang Li, Lina Xiao

**Affiliations:** 1 College of Ocean Engineering Equipment, Zhejiang Ocean University, Zhoushan 316022, China; quwentao2026@163.com (W.Q.); xiaohuang@zjou.edu.cn (X.H.); qiangli@zjou.edu.cn (Q.L.); xiaolina@zjou.edu.cn (L.X.); 2 School of Civil Engineering, Fuzhou University, Fuzhou 350108, China; 18811965070@163.com; 3 Key Laboratory of Nearshore Engineering Environment and Ecological Security of Zhejiang Province, Zhoushan 316022, China

**Keywords:** cement concrete pavement, heavy load, acceleration, dynamic stress, strain energy density

## Abstract

Cement concrete is a critical pavement construction material. However, under prolonged exposure to heavy traffic loads and the combined effects of multiple factors, it frequently exhibits premature slab failure and concurrent multiple defects, severely limiting its service performance and lifespan. The dynamic behavior of cement concrete pavement under heavy-load conditions and the influence of subgrade geometry and pavement width on dynamic bearing performance remain insufficiently understood. To address this issue, this study employs finite element software Abaqus 2020 to construct a three-dimensional finite element model of heavy-duty cement concrete pavement under six typical conditions, including uncut and unfilled subgrade, low embankment, high embankment, cut slope, and different pavement widths. Utilizing an implicit dynamic algorithm, the model simulates and analyzes the acceleration response, dynamic stress distribution, and evolution of strain energy density within the pavement structure under vehicle dynamic loading. The results indicate that the peak acceleration is highest for the uncut subgrade (159.214 m/s^2^) and lowest under the cut condition (146.566 m/s^2^), demonstrating that the cutting structure can effectively suppress pavement vibration intensity. Among subgrade types, high embankments exhibit the greatest capacity for reducing strain energy concentration; at the slab corners, the baseline strain energy density of 8.882 J/m^3^ is reduced by 4.2%, 7.8%, and 5.0% under low embankment, high embankment, and cut conditions, respectively. Regarding pavement width, wider configurations reduce slab vibration intensities, stored strain energy, peak stresses, and stress concentrations, benefiting long-term service life, but concurrently elevate slab corner strain energy accumulation, increasing the risk of corner fracture and compromising load-bearing capacity. These findings provide scientific and technical support for the structural design and performance optimization of heavy-duty cement concrete pavements.

## 1. Introduction

With the steady advancement of economic globalization, heavy-duty transportation has become a core medium supporting the cross-regional circulation of bulk commodities such as energy, leveraging its advantages of efficient and high-capacity material transport. It plays an irreplaceable strategic role in promoting the coordinated development of regional economies. However, the widespread occurrence of axle loads exceeding design standards, high-frequency repetitive loading, and the complex dynamic load characteristics of heavy-duty vehicles pose severe challenges to the long-term service life of road infrastructure [1,2]. These conditions cause significant damage to cement concrete pavements, which are widely used in high-grade highways and industrial park roads. Such damage reduces road smoothness and durability, potentially causing vehicle instability during operation [3], which increases the risk of traffic accidents [4,5].

Although cement concrete pavement has significant advantages, such as high strength, strong stability, and low maintenance costs, under the long-term continuous action of heavy-load traffic, its actual service life can be much shorter than the design expectation, and typical pavement distresses, such as slab fracture and joint misalignment, often occur prematurely. This not only increases the economic cost and resource consumption associated with road maintenance and repair, but also reduces the traffic efficiency of the road network, and may even induce potential traffic safety hazards, thereby restricting the sustainable development of heavy-load transportation systems.

In order to improve pavement performance, many studies have conducted research through multi-scale experimental methods, micro-level analysis, and intelligent information acquisition techniques [6,7,8,9,10]. Regarding structural failure mechanisms, Xue et al. [11] analyzed the fatigue damage evolution of cement concrete pavements under traffic loading using the fully coupled method. Focusing on critical structural components, Zhou et al. [12] and Tan et al. [13] investigated the damage-plasticity and ultimate bearing capacity of the dowel bar systems in cement concrete pavements, where Zhou et al. [12] employed Abaqus software and found that the ultimate capacity, which was around 1.75 times the concrete tensile strength, was reached during the damage and cracking stage of bottom concrete, while Tan et al. [13] combined the extended finite element method with sub-model technology to accurately simulates the ultimate bearing capacity of the dowel bars under dynamic loads. Furthermore, studies by Hong et al. [14] and Maitra et al. [15] highlighted how structural and material parameters, such as the side length of precast block pavements, the elastic modulus of the panel, and the resilient modulus of the soil base, significantly affect the bearing performance and crack evolutions of concrete pavements. In general, these studies established a robust understanding of material-level limits and localized distress mechanisms, providing a necessary baseline for structural health assessment.

Building upon these foundations, recent studies have increasingly addressed the complex dynamic interactions between vehicles and pavements. Wang [16] and Gong et al. [17] employed numerical simulations to demonstrate how dynamic loadings and tire-structure interactions exacerbate pavement distresses and fatigue. Methodological diversification has further refined these characterizations. For instance, Patil et al. [18] introduced artificial boundaries to divide the subgrade into finite and infinite domains; they also proposed influencing parameters for the dynamic response of pavement with and without vehicle–pavement coupling. Alisjahbana et al. [19] quantified the effects of vehicle speed and overloading on the dynamic response of semi-rigid base asphalt pavement. Wang et al. [20] adopted an advanced three-dimensional finite element model to simulate the pavement response and tire contact stress under non-uniform dynamic loads. Additionally, Koh et al. [21] evaluated structural fatigue under superloads, while Xiao et al. [22] studied pavement lateral uneven settlements through combining centrifugal model test with finite element analysis. To bridge the gap between simulation and reality, Wu et al. [23] utilized accelerated pavement testing to assess the bearing capacity and structural performance of concrete pavements under accelerated loadings. Collectively, these works transition the research focus from stationary load limits to the time-dependent behavior of pavement systems under moving traffic.

In summary, the previous research has extensively analyzed pavement dynamics through analytical, numerical and experimental lenses [24,25,26]. However, the integration between different research approaches remains limited, and the obtained results are often difficult to directly apply in practical engineering projects. In addition, most existing studies primarily focused on static mechanical responses, while the dynamic behavior of pavement structure under heavy load conditions has not been sufficiently investigated. In particular, the relationships among dynamic stress, vibration evolution, and strain energy accumulation remain insufficiently understood. Furthermore, the influence of key structural parameters, such as subgrade form and road width, on the dynamic bearing performance of pavements has not been systematically clarified, which makes it difficult to provide reliable theoretical guidance for pavement structure design under heavy-load conditions.

To address the aforementioned gaps, this study investigates the following key questions: (1) how do different subgrade configurations influence the dynamic stress response, vibration behavior, and strain energy accumulation of heavy-duty cement concrete pavement? (2) what competing mechanical effects does pavement width variation introduce on dynamic bearing performance? (3) what are the governing mechanisms linking these structural parameters to pavement fatigue and fracture risk under heavy-load conditions? To tackle these questions, this paper establishes a numerical model using Abaqus finite element software to analyze the dynamic bearing capacity of the pavement. Focusing on the dynamic stress response, structural vibration characteristics, and energy propagation within the pavement structure under heavy-load conditions, this study conducts an in-depth analysis of the mechanisms inducing pavement fatigue damage and impact fracture, and further clarifies the influence of subgrade form and road width on the dynamic bearing performance of the pavement structure. The findings are intended to provide a numerical basis and qualitative guidance for the dynamic design of heavy-duty cement concrete pavements and to identify priorities for improving the bearing performance and service life of pavement structures under heavy-load conditions.

## 2. Materials and Methods

### 2.1. General Methodology and Working Conditions

In this research, all simulations were conducted using Abaqus 2020 (Dassault Systèmes, Providence, RI, USA), a commercially licensed finite element software package widely adopted in pavement engineering research [27,28]. Based on the Abaqus platform, a three-dimensional finite element model of a heavy-duty cement concrete pavement is established. By observing the variation in the subgrade response beneath the pavement slab with the position of the vehicle dynamic load, the influence of subgrade shape and road width configuration on the dynamic bearing performance of the pavement structure can be investigated. The working conditions considered are summarized in Table 1.

### 2.2. Model Dimensions

**Figure 1:**
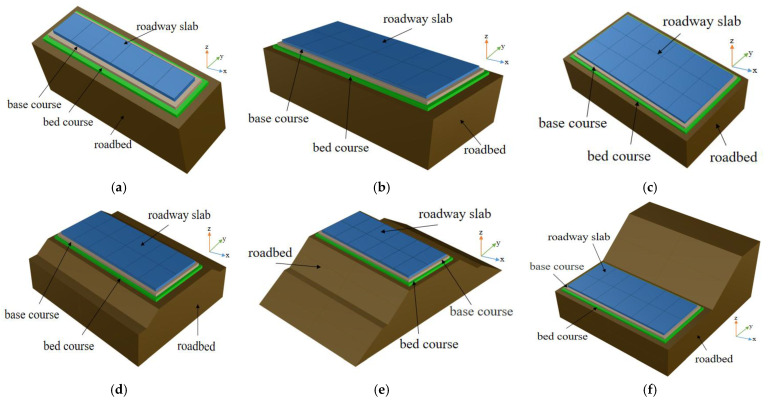
Typical finite element models of cement concrete pavement. (**a**) Unfilled excavation roadbed-single pavement model; (**b**) Unfilled excavation roadbed-double pavement model; (**c**) Unfilled excavation roadbed-three pavement model; (**d**) Low embankment-double pavement model; (**e**) High embankment-double pavement model; (**f**) Cut-double pavement model.

To observe the influence of the surrounding environment on the dynamic bearing capacity of the heavy-duty cement concrete pavement, the finite element model of the five-plate pavement structure is established, with the middle plate designated as the observation plate. The detailed structural dimensions of each model are shown in Table 2. The three-dimensional finite element model of each structure presented in this study is shown in Figure 1.

### 2.3. Parameter Settings

#### 2.3.1. Material Parameters

According to the characteristics of heavy-duty traffic scenarios, the pavement structure model adopts a four-layer system, consisting from top to bottom of concrete surface course, base course, subbase, and soil subgrade. The physical and mechanical parameters of each layer are listed in Table 3. This model is also designated to satisfy the following basic assumptions and modeling conditions: (1) each structure is a uniform, continuous, and isotropic elastomer; (2) the interlayer contact is fully continuous; (3) to simplify the model, dowel bars and tie bars are not considered, no joint-filling material is included, and no load transfer occurs across the joints; (4) the structure considers self-weight.

It should be noted that dowel bars and tie bars are deliberately excluded to represent the most unfavorable load transfer condition, consistent with conservative engineering safety evaluation principles. This approach amplifies stress and energy accumulation at joints and panel edges, enabling more effective identification of potential crack initiation locations and structural weak points. Moreover, while these material assumptions may produce idealized absolute magnitude predictions, their influence on relative trends between configurations is limited as all working conditions share an identical modeling framework.

#### 2.3.2. Load Parameters

**Figure 2:**
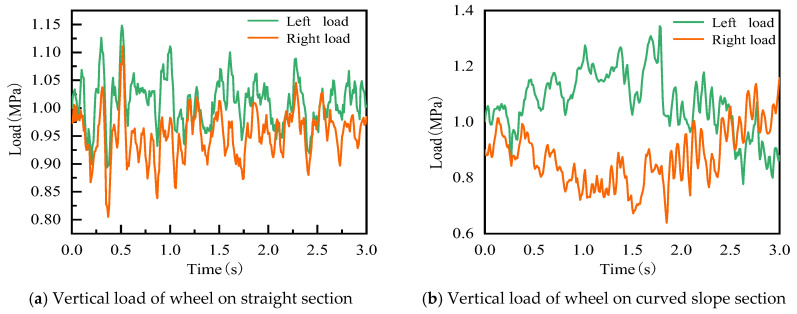
Time histories of vehicle inertia loadings.

In this paper, based on the six-component field vehicle load experiment, it is found that the vehicle exerts inertial loads on the road surface during their operation, especially in the special road sections (bending and slope section). In addition, since the inertial load changes significantly with the line type and wheel, the inertial load of the vehicle under the straight line and the curved slope is selected separately to analyze the influence of the line type, as shown in Figure 2. In the Abaqus calculation, the moving inertial load is applied using a self-compiled VDLOAD subroutine; the left and right wheels experience different inertial loads, which is mainly achieved by adding code to the INP file and combining it with the VDLOAD subroutine.

#### 2.3.3. Load Positions

**Figure 3:**
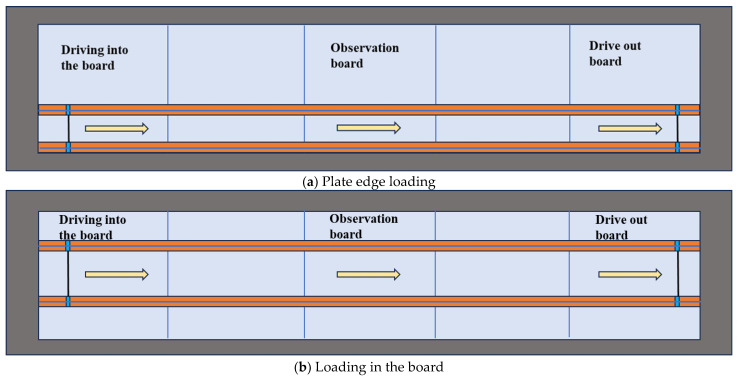
Loading positions.

The long-side half of a single road surface is considered the most unfavorable position [29]; however, most vehicles are actually found to travel across the plate. Therefore, this study applies the vehicle load at both the most unfavorable position and the plate, as shown in Figure 3.

#### 2.3.4. Boundary Constraints

**Figure 4:**
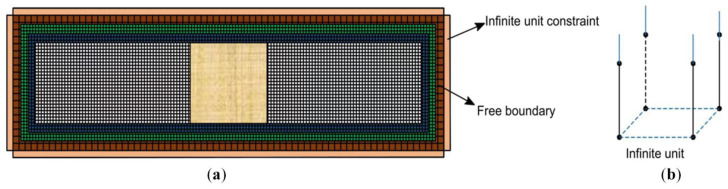
(**a**) Model constraint diagram; (**b**) CIN3D8: 8-node linear unidirectional infinite element.

In order to simplify the calculation model, the tie constraint is selected [30,31]. However, this constraint cannot account for slip or debonding of the pavement slab, which leads to the deterioration of the bearing capacity of the pavement. Therefore, the tie constraint is adopted between the base layer, the cushion and the soil base, while surface-to-surface contact is applied between the surface layer and the base layer. The surface-to-surface contact includes two aspects: the normal contact is set to hard contact to prevent penetration during the calculation [32], and the tangential contact is set to frictional contact with a friction coefficient of 0.6. Considering that in practice the road surface exhibits back and forth oscillation of stress wave, like the effect of an infinite boundary model, the present model adopts infinite boundary constraints. That is, the soil base is modeled using infinite element constraints, while the rest of the structure is free at its boundaries, as illustrated in Figure 4a. In this paper, a layer of infinite elements (CIN3D8) is applied to its lateral sides and bottom in the finite element model, thereby providing proper connection of these boundaries.

#### 2.3.5. Model Validation

**Figure 5:**
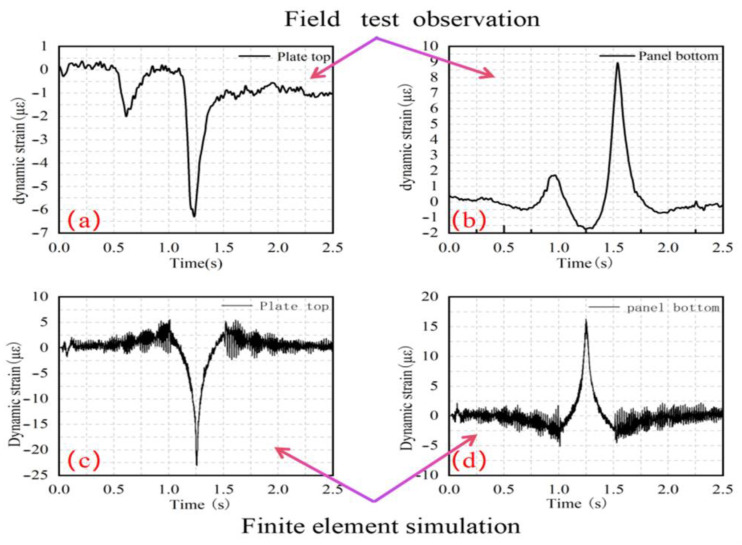
Measured dynamic strain time histories at (**a**) slab top and (**b**) slab bottom, and simulated strain time histories at (**c**) slab top and (**d**) slab bottom.

To verify the reliability of the finite element model, field measurements were carried out on a test road section. In the test, a rear single-axle vehicle was driven at a speed of 10 m/s, and the dynamic strain responses at the top and bottom of the pavement slab were collected through the Wheel Force Transducers (WFTs) manufactured by Michigan Scientific Corporation, MI, USA. The measured results are shown in Figure 5a,b, respectively. It can be seen from the finite element simulation results shown in Figure 5c,d that the dynamic strains at the top and bottom of the slab are symmetrically distributed vertically, with alternating compressive and tensile strains. The top of the slab is mainly under compression, while the bottom of the slab is mainly under tension. This pattern is consistent with the measured results. In terms of waveform characteristics, the overall measured strain curve is relatively smooth with only minor fluctuations, whereas the strain curve obtained by finite element calculation shows obvious zigzag fluctuations when the vehicle enters and leaves the observation slab. This is mainly because the propagation, reflection, transmission and superposition effects of the stress wave caused by the vehicle load in the pavement structure are considered in the simulation. When the sampling frequency of the measurement system is high enough, similar high-frequency fluctuation components can also be captured.

Comparing the peak strains, the measured peak strains at the top and bottom of the slab are approximately 6 με and 9 με, respectively, while the simulated peak strains at the top and bottom of the slab are 23 με and 16 με, respectively, which are 17 με and 7 με higher than the measured values, respectively. This discrepancy is acknowledged and can be attributed to three identifiable factors. First, the model adopts an idealized homogeneous subgrade, fixed boundary constraints, and standardized dynamic load input, whereas field conditions involve subgrade non-uniformity, nonlinear interlayer contact behavior, and inherent vehicle load randomness that cannot be fully reproduced in the numerical framework; this boundary condition mismatch is considered the primary contributor to the deviation. Second, the model employs static mechanical parameters for the concrete surface layer and base course without accounting for strain-rate-dependent material behavior under dynamic loading, which can contribute to strain overestimation. Third, field strain measurements are subject to temperature drift in embedded gauges, local stress concentrations near joints, and lateral deviation of vehicle wheel tracking; additionally, an inherent dimensional difference exists between point-measured field strains and the spatially averaged element strains output by the finite element model, meaning that a direct peak-value comparison does not fully represent the true degree of model-measurement agreement.

It is important to note that all primary conclusions of this study are based on relative comparisons between different subgrade types and pavement width configurations evaluated within a consistent modeling framework, rather than on absolute strain magnitudes. The systematic overestimation identified above affects all working conditions equally and therefore does not alter the relative trends or percentage differences reported between conditions. The model reliably reproduces the qualitative dynamic response characteristics of the pavement structure, including stress distribution patterns, vibration behavior, and energy propagation mechanisms, and is therefore considered suitable as the basis for the comparative analyses conducted in subsequent sections. It is also acknowledged that formal uncertainty quantification and sensitivity analysis of key parameters such as subgrade modulus and material damping are not conducted in this study and are recommended as a direction for future research.

## 3. Results and Discussion

### 3.1. Characterization of Dynamic Bearing Performance of Pavement Structure Under Heavy Load

The dynamic bearing capacity of pavement refers to its ability to resist deformation and damage under vehicle loads. This capacity is reflected in the periodic evolution of dynamic stress distribution, vibration response, and energy propagation and dissipation within the pavement slab under vehicle dynamic loads. This study focuses on the dynamic behavior of pavement structure under heavy traffic conditions to investigate its dynamic bearing performance. The pavement’s dynamic bearing capacity is affected by multiple dynamic behaviors under traffic load, including dynamic responses, fatigue accumulation, damage evolution, and impact fracture. Currently, most researchers evaluate fatigue and damage in cement concrete pavements based on fatigue damage accumulation theory, which uses the S-N curve to evaluate the bearing capacity under repeated loads. For impact fracture, fracture mechanics theory is used to analyze crack resistance and failure mechanisms from the perspective of crack initiation and irregular propagation. Overall, it can be seen that starting from the dynamic response, vibration characteristics and energy evolution law of pavement structure under heavy load can effectively reveal the patterns and deterioration mechanisms of its dynamic bearing performance.

### 3.2. Analysis of Dynamic Bearing Capacity of Cement Concrete Pavement Under Heavy Load Traffic

#### 3.2.1. Dynamic Stress Response Analysis

**Figure 6:**
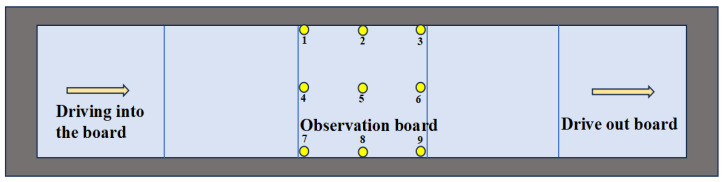
Layout of observation points used to evaluate the dynamic bearing capacity of the heavy-duty traffic cement concrete pavement.

**Figure 7:**
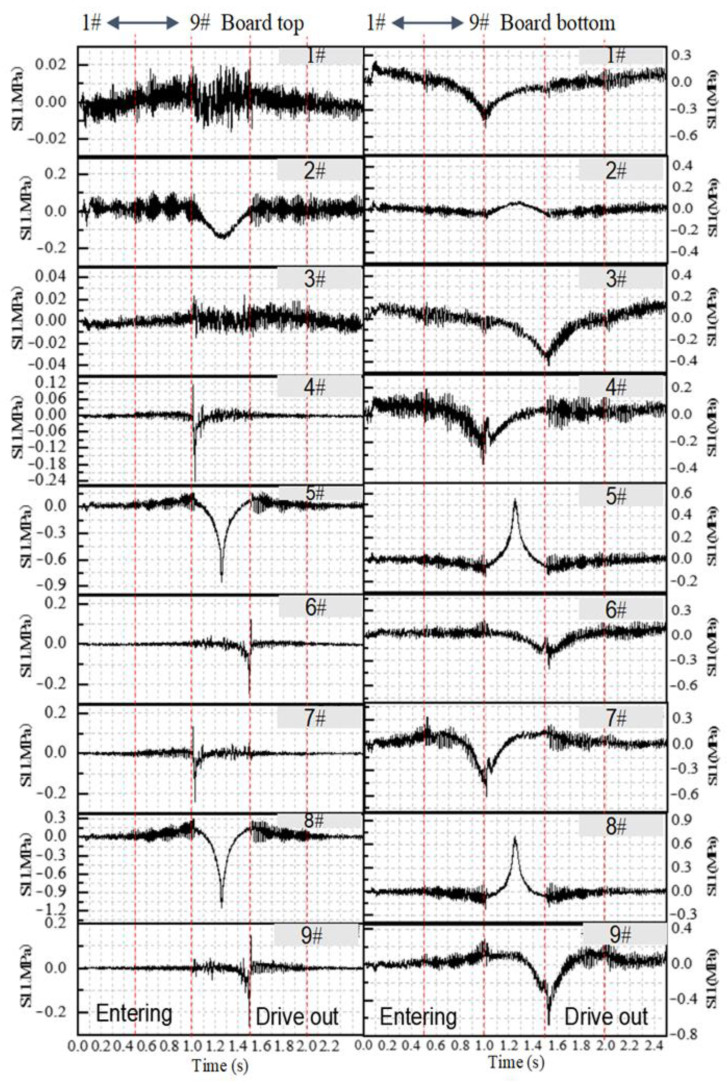
Road panel dynamic stress matrix diagram.

Based on the three-dimensional finite element model described in Section 2, i.e., adopting the single-width unfilled subgrade configuration under plate edge loading, with material parameters as listed in Table 3 and vehicle loading applied via the VDLOAD subroutine, the vehicle travels at a speed of 10 m/s from entering to exiting the road. According to Figure 6, the dynamic stress at the top and bottom of the observation plate at nine positions is extracted. Figure 7 shows the dynamic stress time history matrix of these observation points when the vehicle moves to different positions. It can be seen from Figure 7 that the top and bottom of the plate are subjected to alternating tensile and compressive stresses, with the top of the plate mainly under compression, and the bottom of the plate experiencing both tensile and compressive stress. The dynamic stress on the top of the plate initially increases and then decreases with the change of the position of the vehicle load, reaching a peak when the load is applied to the lateral position of the observation point. The peak value of the compressive stress at the top of the plate is 1.15 MPa. Observation points 2#, 5# and 8# at the bottom of the plate are subjected to tensile stress, while the other observation points experience compressive stress. The peak tensile stress is 0.71 MPa, and the peak compressive stress is 0.22 MPa.

When the vehicle load is applied either in front of or behind the observation plate, the plate experiences stress fluctuations of alternating tension and compression. The top stress of the long-side plate fluctuates the most, while the bottom stress of the short-side plate fluctuates significantly and increases noticeably compared with the top of the plate. Since the panel joints do not transmit load, this indicates that the dynamic stress from distant vehicle loads is transmitted to the base layer, and the observed pavement panel is affected in the form of stress waves. From the vehicle load to the position of the observation point, it can be seen that, unlike the top of the plate, the stress peak at the bottom of the plate is superimposed with jagged fluctuations, which are the result of the superposition of environmental vibration and stress wave reflection at the surface layer and the base layer. Although the stress magnitude is small, the fluctuation frequency is large, the duration is long, and the tension and compression alternate repeatedly. Although this will not adversely affect the pavement plate structure, it can easily damage the cement-aggregate bonding surface, produce fine cracks, cause material deterioration, and create unfavorable zones.

**Figure 8:**
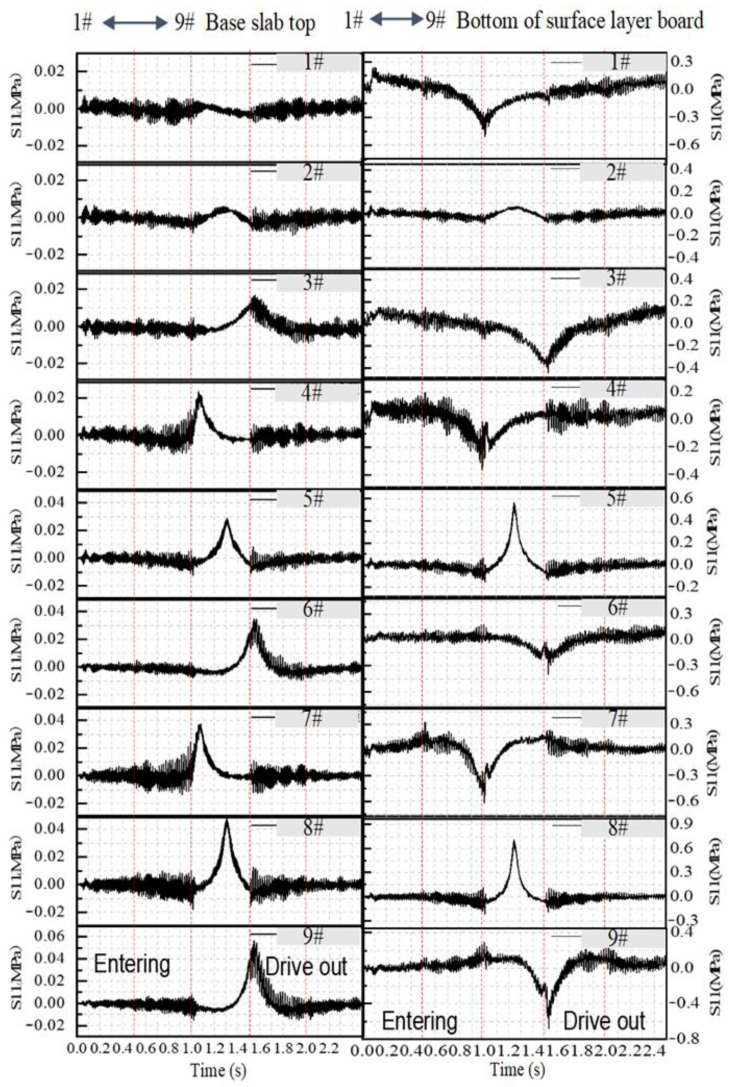
Dynamic stress matrix diagram of base and surface layer.

In order to reveal the generation of stress waves when the observation plate is unloaded, the longitudinal dynamic stress time history curves at the bottom of the surface layer and at the foundation surface layer are extracted, and the dynamic stress matrix diagram is plotted in Figure 8. It can be seen that the longitudinal dynamic stress at the top of the base is tensile. The tensile stress at the top of the plate changes with the position of the load, first increasing and then decreasing, and reaches its peak at the observation point. The maximum tensile stress is 0.06 MPa, which is significantly lower than that of the surface layer. When the vehicle is immediately in front of or just beyond the observation plate, although the dynamic stress time history still fluctuates, the fluctuation range is much smaller than that at the bottom of the plate. When the axial load acts on the observation plate, the fluctuation range of the base stress is further reduced. The distant load is not transmitted through the base layer, but mainly propagates in the form of stress waves, and the stress is significantly mitigated when it is reaches the base layer.

#### 3.2.2. Vibration Behavior Analysis of Pavement Structure Under Heavy Load

**Figure 9:**
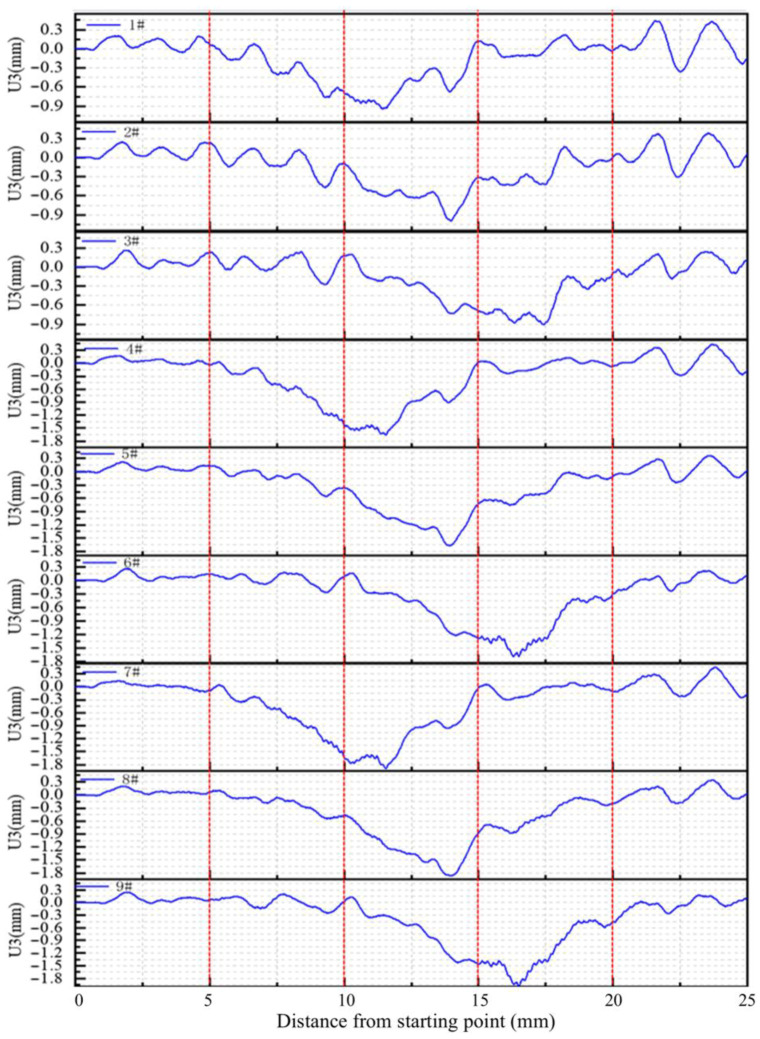
Deflection–distance curve of the pavement.

When the vehicle moving load acts on the road surface, the deflection value of the road surface is negative, which indicates that the panel exhibits reverse vibration behavior. To observe these phenomena more clearly, the displacement time history at each observation point as the vehicle travels over different positions under biaxial load is extracted, and the pavement’s deflection curves are plotted in Figure 9. It can be seen from Figure 9 that when the vehicle is driving on the first and the last boards, the observation board as a whole does not show a downward trend, but fluctuates around zero, with observation points 1#, 2# and 3# showing the most pronounced fluctuations. This indicates that under the action of vehicle load, the observation plate exhibits reverse vibrations, and due to the asymmetry of the loading position, a significant reverse bounce occurs at the opposite position of the load, reaching up to 0.38 mm. Therefore, when the heavy load acts on the edge of the plate, the symmetrical interface is prone to debonding, which affects the service life of the pavement.

As the vehicle moves across the plates, the deflection values of observation points increase sequentially: points 1#, 4# and 7# began to increase when the vehicle just enters the second plate; points 2#, 5# and 8# gradually increase when it enters the second plate; points 3#, 6# and 9# began to increase when it enters the observation plate. By comparing the positions of the observation point, it can be found that when the load is 5 m away from the observation point, the deflection value begins to increase. As the vehicle moves further, the deflection value reaches a peak, and then gradually decreases. This trend shows that loads beyond 5 m have little effect on the vibration of the observation point, which can be regarded as environmental vibration. The occurrence of reverse bounce vibration may be due to the inconsistency of the stiffness radius between the pavement and the subgrade. The effect of distant load on pavement structure is transmitted through base vibration. Combined with the above analysis, it can be found that environmental vibration produces stress waves.

Notably, these vibration characteristics directly relate to the pavement’s resonant frequencies, which are central indicators in structural health monitoring (SHM) systems [33]. Higher acceleration peaks, uneven dynamic stress distributions, and elevated strain energy densities can all reduce the fundamental resonant frequency and increase the risk of resonance-induced fatigue. For SHM applications, accelerometers are well suited to capturing such global vibration responses due to their high sensitivity and ease of deployment, whereas tensometers (strain gauges) provide localized strain information but require careful installation and compensation. A combined use of both sensor types can enhance the reliability of pavement performance evaluation.

#### 3.2.3. Analysis of Energy Behavior of Pavement Structure Under Heavy Load

There are energy storage, propagation and release processes in the pavement structure under vehicle load. When the energy at a defect position reaches a critical point, energy release behaviors such as material crack propagation and structural fracture occur. The distribution of energy reflects how energy propagates and is stored in the structure. In this study, the term “energy storage” refers specifically to the elastic strain energy stored within the pavement structure (including the slab, base, subgrade, and joint regions) due to elastic deformation under dynamic heavy vehicle loading. It excludes energy dissipated through plastic deformation or material damage. This metric serves as a core indicator for characterizing the dynamic bearing capacity and identifying potential weak links, as energy accumulation at specific locations is directly associated with an elevated risk of crack initiation and propagation. Based on the linear elastic numerical model established in this study, the elastic strain energy is rigorously quantified using the standard formula for elastic strain energy density integrated over the element volume:

(1) $$U=\frac{1}{2}\sigma \varepsilon V$$

where *U* is the elastic strain energy stored in a finite element (unit: J), *σ* is the maximum principal stress (unit: Pa), *ε* is the corresponding dynamic peak strain (dimensionless), and *V* is the element volume (unit: m^3^). The total energy storage at a given location is obtained by summing the contributions of elements within the region of interest.

**Figure 10:**
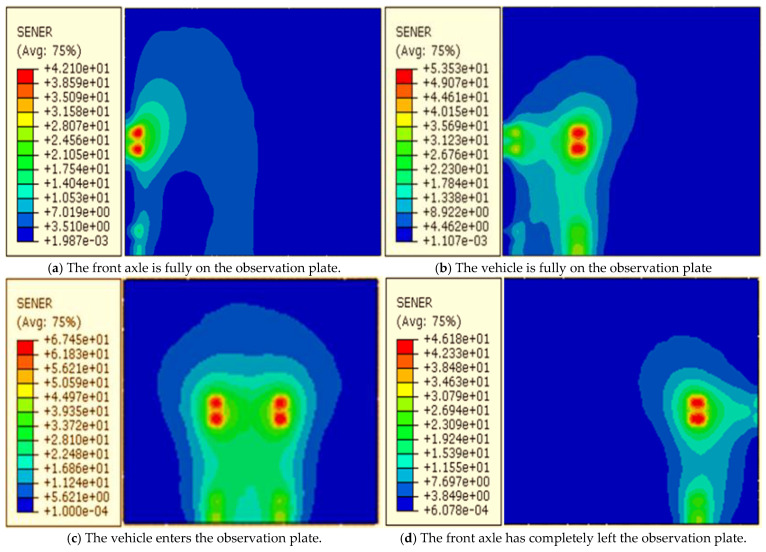
Strain energy density distribution nephogram of the plate top at different vehicle positions.

To explore the energy distribution in the pavement structure, the strain energy density cloud diagram of the slab top under biaxial load is extracted. From Figure 10a–d, it can be seen that the strain energy concentration changes with the position of the external force. At the load application position, the energy diffuses outward in a wavy manner and is irregular due to the superposition of multi-point diffusion waves. Among them, the energy density at the loading position is the largest, reaching up to 67.5 J/m^3^. On the whole, the strain energy distribution is asymmetric. The energy density under the left wheel is large, while that under the right wheel is smaller, which may be related to the asymmetry of the left and right wheel loads of the vehicle. There are energy concentration, diffusion and superposition in the horizontal direction, and the energy concentration region is located at the load application area. It should be noted that in actual conditions, energy storage in the structure may occur due to the high frequency of vehicle loading, which can ultimately cause premature damage to the structure.

**Figure 11:**
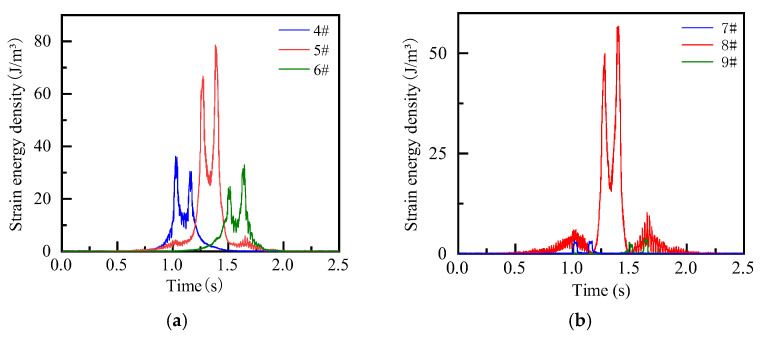
Strain energy density time history curves at different positions on the top of the plate. (**a**) Strain energy density time histories at the middle of the plate; (**b**) strain energy density time histories at the edge of the plate.

In order to reveal the propagation of energy in the pavement structure, the vertical propagation pattern is analyzed by taking the vehicle driving onto the observation plate as an example. The strain energy density at the top of the road surface is large, and it prone to concentration. At the joints of the road surface, due to the frequent action of the vehicle and the low smoothness of the joint, stress damage is relatively severe. When the concentrated energy reaches a critical value, it can cause the initiation and expansion of the longitudinal joint until the panel fails. At the same time, from the overall analysis, the energy is also transmitted vertically downward as fluctuations from the point of action of the external force, and finally dissipates in the soil foundation. It is worth mentioning that large energy concentration is also prone to occur at the interface between the surface layer and the base layer, and it will superimpose at the bottom of the base layer under multi-axial conditions, which is defined as the energy storage behavior of the structure in this paper. When energy storage reaches a critical value or the road surface deteriorates to a critical point, bottom-up road damage occurs. The analysis shows that the strain energy density along the driving track is relatively high. To further analyze the relationship between the strain energy density of the observation plate and time, the density time histories of observation points 4#–9# are extracted. It can be seen from Figure 11 that there is a large energy concentration at the joint, and even under multi-axis loading, as the energy from the front axle is not fully dissipated, energy superposition occurs, and lasts longer, which further confirms that the joint is prone to energy storage behavior and longitudinal seam phenomenon. At the same time, it is also observed that significant energy concentration occurs in the wheel track line, but the rear peak in the plate is smaller than the previous peak and lasts for a shorter time, indicating that the energy in the plate is dissipated relatively quickly. When the vehicle passes from the edge of the plate, the strain energy density at the long-side plate position is the largest, up to 56.7 J/m^3^, far exceeding the plate angle, and a double peak phenomenon is observed. This shows that when vehicles frequently pass along the edge of the plate, transverse cracks are easily formed.

### 3.3. The Influence of Subgrade on the Dynamic Bearing Capacity of Cement Concrete Pavement Structure

**Figure 12:**
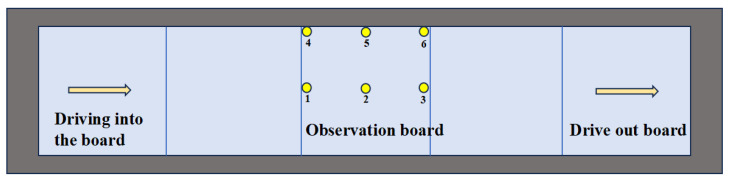
Observation points for impact analysis.

In the process of development and evolution, pavement distresses that affect the dynamic bearing capacity of the pavement show a diversion phenomenon depending on subgrade conditions. Therefore, this paper selects a variety of roadbed types to study the influence of different subgrades on the pavement’s bearing capacity by analyzing their dynamic responses and strain energy density. Figure 12 shows the selected observation points.

#### 3.3.1. The Influence of Subgrade on the Vibration Acceleration of Pavement Structure

**Figure 13:**
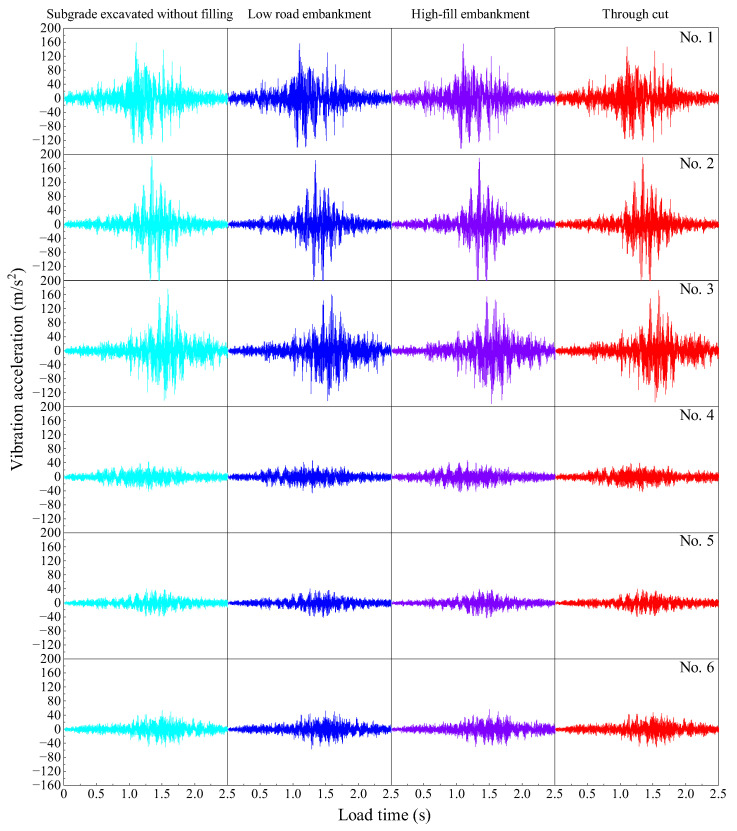
Acceleration time histories at the observation points under different subgrade conditions.

The vibration acceleration of each observation point under different subgrade conditions is calculated for four types of subgrades: no filling, low embankment, high embankment and cutting. Figure 13 shows the vibration acceleration curves of each observation point under these subgrade conditions. It can be seen that when the vehicle enters the driving plate, the acceleration of the road surface increases continuously and reaches a peak near the observation points. This indicates that the heavy load has a significant impact on the road surface vibration, which in turn accelerates fatigue, especially at the material interface between structural layers and the applied load. Taking observation point 1# as an example, the acceleration peaks under the conditions of unfilled and excavated roadbed, low embankment, high embankment and cutting are 159.214 m/s^2^, 156.693 m/s^2^, 154.941 m/s^2^ and 146.566 m/s^2^, respectively. The peak acceleration is lowest under the cutting condition and highest for the unfilled and excavated roadbed, with reductions of 1.6%, 2.7% and 7.9% for low embankment, high embankment, and cutting, respectively, compared with the unfilled and excavated roadbed. This demonstrates that cutting can significantly reduce road surface vibrations and improve the fatigue resistance of the pavement structures.

#### 3.3.2. Influence of Subgrade on Longitudinal Dynamic Stress of Pavement Structure

**Figure 14:**
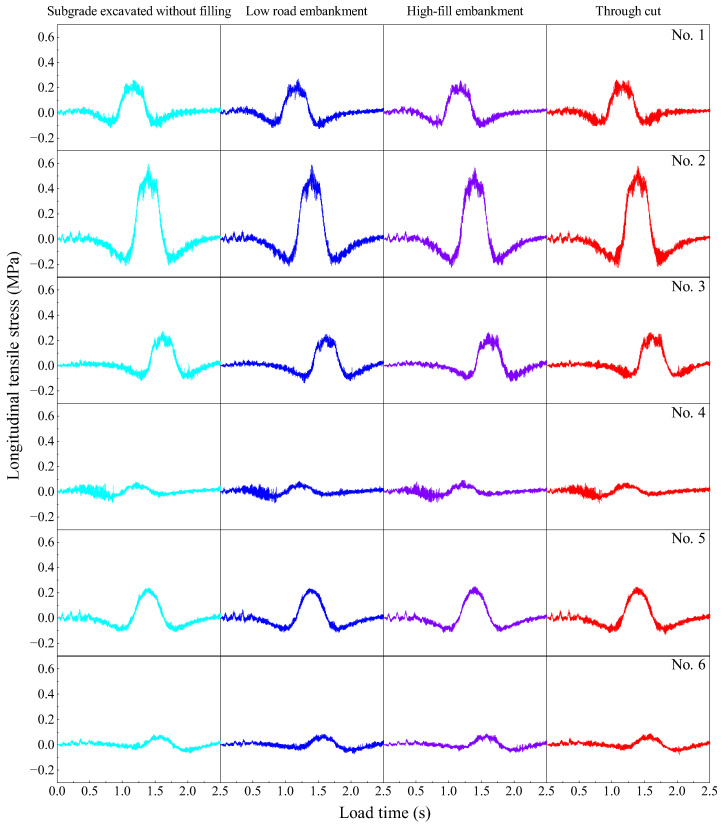
Dynamic stress time histories at the observation points under different subgrade conditions.

From Figure 14, it can be seen that the stress at each observation point exhibits obvious oscillations, and as the load continuously approaches, alternating tensile and compressive stresses occur. This is because the dynamic stress generated in the pavement structure under heavy load propagates, reflects and superposes within the structure in the form of stress waves. The continuous fluctuation of these stresses can easily damage the bonding surface between cement and aggregate, resulting in fine cracks, material degradation, and a reduction in the bearing capacity of the pavement. Comparative analysis shows that the stress at observation point 2# is more pronounced; therefore, observation point 2# is selected to analyze the influence of different subgrade conditions on the dynamic response of the pavement structure. Under these conditions, the stress peaks for the non-filled subgrade, low embankment, high embankment, and cutting are 0.600 MPa, 0.590 MPa, 0.568 MPa, and 0.580 MPa, respectively. It can be seen that a high embankment condition can significantly reduce pavement stress and improve pavement fatigue performance. This indicates that the use of high embankment or cutting conditions can mitigate interface deterioration caused by stress fluctuations in the pavement and improve the dynamic bearing performance.

#### 3.3.3. Influence of Subgrade on Strain Energy Density of Pavement Structure

**Figure 15:**
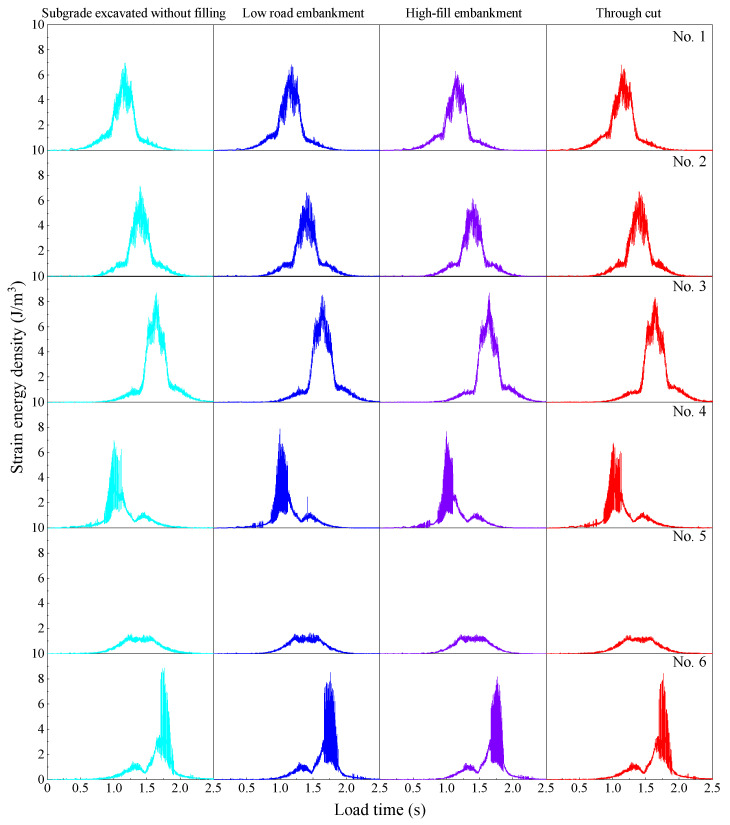
Strain energy density time histories at the observation points under different subgrade conditions.

It can be seen from Figure 15 that the strain energy density at the observation point changes with the vehicle load and the position of the observation point, showing a trend of first increasing and then decreasing, and reaching a peak at the observation point. In addition, compared with the triangular trend observed at the observation points at the wheel trace, 4# and 6# show a sudden increase in energy, and their energy peaks are not lower than those of the observation points at the loading point or even higher than that of the loading point. This shows that there is energy storage on the road surface under heavy load, and the most unfavorable position is not necessarily the wheel track line but may occur at locations such as the corner, which will increase the risk of pavement fracture. Under different subgrade conditions, the plate angle is an unfavorable position for energy concentration. The strain energy density at the corner of the plate is 8.882 J/m^3^, 8.513 J/m^3^, 8.191 J/m^3^, and 8.435 J/m^3^, respectively, under the conditions of unfilled subgrade, low embankment, high embankment, and cutting. The analysis shows that compared with the unfilled subgrade, the values for the low embankment, high embankment, and cutting conditions are reduced by 4.2%, 7.8%, and 5.0%, respectively. This shows that compared with the unfilled subgrade, the other subgrade types significantly reduce the strain energy concentration of the pavement structure under heavy load, thereby reducing the risk of pavement fracture and improving the dynamic bearing performance of the pavement. Among them, the high embankment shows a particularly significant effect in reducing the strain energy concentration of the pavement under heavy traffic.

### 3.4. The Influence of Road Width on the Dynamic Bearing Capacity of Cement Concrete Pavement Structure

#### 3.4.1. The Influence of Road Amplitudes on the Vibration Acceleration of Pavement Structure

**Figure 16:**
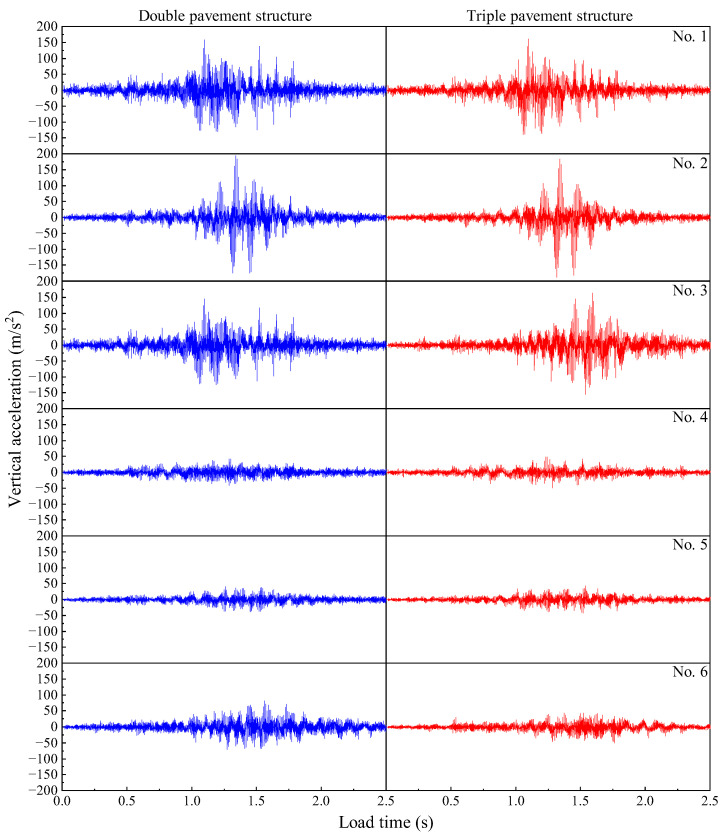
Acceleration time histories at the observation points under different road amplitude conditions.

The double and triple pavement structures were selected to calculate the vibration acceleration of each observation point under different road conditions. The vibration acceleration curves of each observation point under different road conditions are shown in Figure 16. From Figure 16, it can be seen that the vibration acceleration of the road surface under heavy load fluctuates around the “0” line, and increases with the increasing fluctuation of the vehicle load. The vibration acceleration of the observation point at the wheel track line is larger, while that of the observation points away from the wheel track line is smaller, but the vibration acceleration at the corner where the vehicle leaves the end plate is significantly increased. This shows that the departure angle of the end plate is a critical area of vibration, which is prone to fracture, thus affecting the load-bearing performance of the structure.

From the analysis of each observation point, it can be seen that the vibration acceleration of double pavement and triple pavement in 1# is 159.214 m/s^2^ and 161.367 m/s^2^, respectively, 2# is 195.273 m/s^2^ and 183.701 m/s^2^, respectively, 3# is 145.361 m/s^2^ and 163.912 m/s^2^, respectively, and 6# is 82.666 m/s^2^ and 44.246 m/s^2^, respectively. It can be seen that the vibration intensity at the driving end of the double pavement is less than that of the triple pavement, but the vibration intensity within the plate is greater than that of the triple pavement. In addition, the vibration intensity at the corner of the triple pavement is significantly smaller than that of the double pavement. This shows that increasing the road width can reduce the vibration intensity within the plate and at the corners, thereby improving the service life of the pavement, but the increase in the road width may increase the vibration of the pavement joint, which raises the risk of cracking at the joint.

#### 3.4.2. Influence of Road Width on Horizontal Dynamic Stress of Pavement Structure

**Figure 17:**
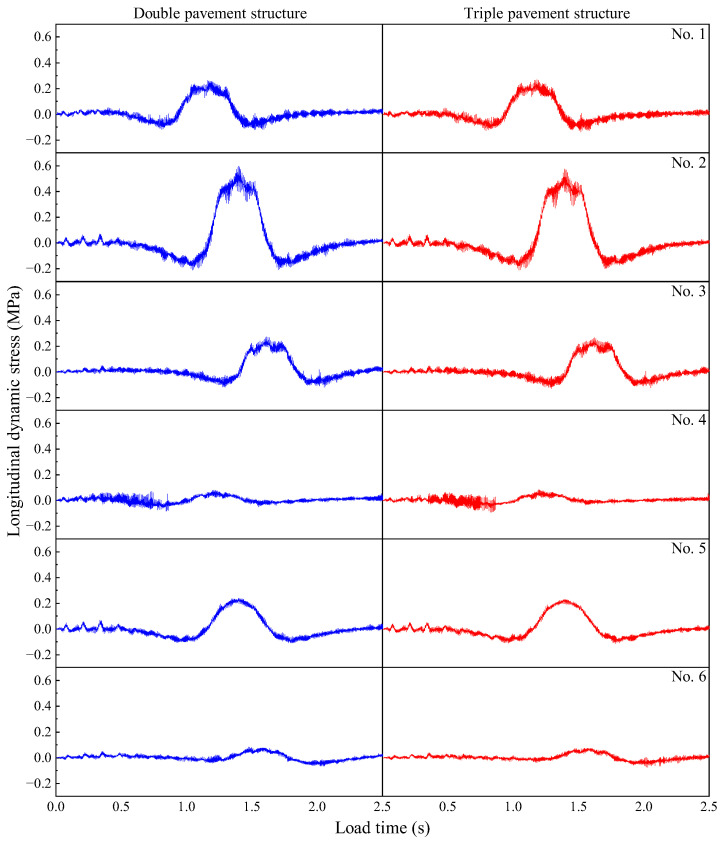
Dynamic stress time histories at the observation points under different road conditions.

It can be seen from Figure 17 that the dynamic stress of the pavement structure is higher in the middle of the plate than at the edge of the plate, and the edge of the plate is higher than the corner of the plate. Among them, the dynamic stress of double pavement and triple pavement at 1# is 0.264 MPa and 0.272 MPa, respectively; at 2# is 0.600 MPa and 0.577 MPa, respectively; at 3# is 0.273 MPa and 0.267 MPa, respectively; and at 5# is 0.242 MPa and 0.232 MPa, respectively. It is found that the dynamic stress of the double pavement is greater than that of the triple pavement, except at the driving end. This shows that increasing the road width can significantly reduce the peak stress and stress concentration of the pavement, but it will increase the force on the pavement structure when driving, thereby increasing the risk of longitudinal cracking at the joint.

#### 3.4.3. Influence of Road Width on Strain Energy Density of Pavement Structure

**Figure 18:**
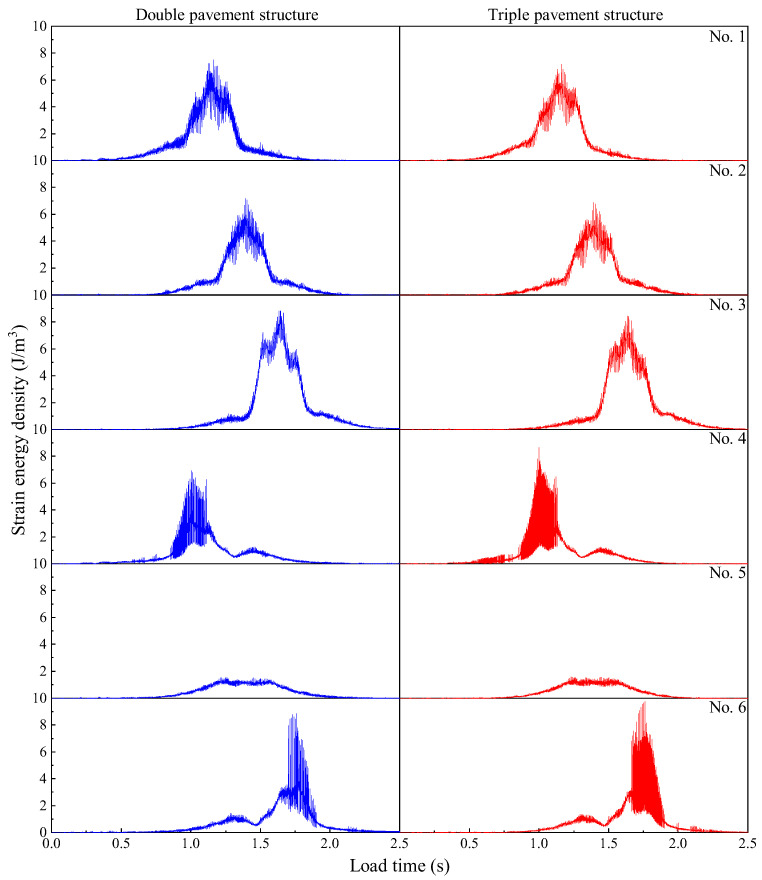
Strain energy density time histories at the observation points under different road conditions.

It can be seen from Figure 18 that when the vehicle gradually enters the observation plate, the energy at the observation points increases continuously, and the energy storage at the wheel track line and the plate angle is the largest, with the energy fluctuation at the plate angle being particularly large. This shows that the energy in the plate increases with the action of the vehicle load, but the energy release at the corner of the plate is faster, while the energy release within the plate is slower, resulting in greater energy accumulation. From the analysis of observation points, the strain energy density of double pavement and triple pavement at 1# is 7.531 J and 7.192 J, respectively; at 2# is 7.186 J and 6.88 J, respectively; at 3# is 8.857 J and 8.473 J, respectively; at 4# is 6.955 J and 8.677 J, respectively; and at 6# is 8.882 J and 9.762 J, respectively. The analysis shows that the strain energy density in the plate decreases with the increase in road width, while the strain energy density at the corner of the plate increases with the increase of road width. This shows that increasing road width can significantly reduce the strain energy stored in the plate, but it is unfavorable for the corner of the plate, which can easily lead to corner failure, thus affecting the load-bearing performance of the pavement.

## 4. Conclusions

This paper investigates the dynamic bearing performance of heavy-duty cement concrete pavement under heavy traffic loading and systematically examines the influence of subgrade type and road width on its structural response through numerical comparative analysis under idealized modeling conditions. The main conclusions are as follows:

(1) Under heavy load conditions, the pavement structure generates various dynamic behaviors including dynamic stress responses, structural vibrations, and energy propagation and release. These dynamic behaviors collectively have the potential to induce fatigue, damage and impact fracture of the pavement, which may further lead to the deterioration of bearing performance and a significant reduction in service life.

(2) Within the modeled configurations, the adoption of high embankment and cutting structures is shown to effectively alleviate interface deterioration caused by stress fluctuations, thereby improving the dynamic bearing performance of the pavement. In particular, cutting structures demonstrate a more pronounced effect in reducing pavement vibration intensity, suggesting greater potential for enhancing the pavement’s fatigue resistance.

(3) Compared with the cut-and-fill-free subgrade, all other subgrade types investigated in this study effectively reduce strain energy concentration under heavy loads, thereby indicating potential for mitigating fracture risk and improving dynamic bearing performance. Among these, high embankments exhibit the greatest effectiveness in suppressing strain energy concentration, with a reduction of 7.8% at the slab corner under the modeled conditions.

(4) Increasing road slab width produces competing mechanical effects. On one hand, it reduces the vibration intensity at the mid-slab and slab corners, lowers the peak stress and stress concentration, and decreases mid-slab strain energy storage, all of which contribute to prolonging pavement service life. On the other hand, it simultaneously exacerbates joint vibration, increases the risk of joint cracking, elevates stress at the entry end, and promotes strain energy accumulation at slab corners, collectively compromising the overall bearing performance of the pavement.

Based on the findings of this study, the following design recommendations are proposed for heavy-duty cement concrete pavements: (1) high embankment or cutting configurations are preferred for heavily trafficked sections, while pavement stiffness should be enhanced for unfilled and excavated subgrade sections; (2) pavement width should be increased strategically, with reinforced structural protection applied to slab corner regions; (3) vibration monitoring using accelerometers and load calibration using dynamometers are recommended during operation, and periodic inspection and timely repair of micro-cracks at panel edges and joint areas should be conducted to prevent progressive deterioration; (4) particular attention should be given to joint design and construction, for instance, load transfer devices such as dowel bars are recommended for heavily trafficked sections to reduce stress and energy accumulation at joints, and sealing treatment should be applied to prevent subgrade erosion from water infiltration.

In terms of limitations, future research should focus on three aspects: (1) further parameter uncertainty quantification and sensitivity analysis are needed to improve the generalizability of these conclusions; (2) investigation should be expanded to cover the coupling effects of environmental factors and heavy-duty traffic loads to advance distress prevention and control theories; (3) long-term field measurements should be carried out to verify the simulation results and support the development of pavement life prediction models.

## Figures and Tables

**Table 1:** Engineering design table.

Research Content		Work Condition	Observed Quantity
Bearing capacity performance of heavy traffic cement concrete pavement		Plate edge loading, single width, no filling excavation embankment	Dynamic stress, displacement and energy
Impact analysis of special effects	Roadbed form	Loading in the board, No filling excavation roadbed, Single width	Dynamic stress, acceleration and energy
		Loading in the board, Low embankment, Single width	
		Loading in the board, High embankment, Single width	
		Loading in the board, Cutting, Single width	
	Road width condition	Loading in the board, No filling excavation roadbed, Single width	
		Loading in the board, No filling excavation roadbed, Double amplitude	
		Loading in the board, No filling excavation roadbed, Three panels	

**Table 2:** The size of each model structure layer (unit: m).

Model Type	Structural Layer	Longitudinal	Transverse	Thickness
Single unfilled excavation roadbed	Concrete surface layer	5.00	4.50	0.24
	Foundation course	26.04	5.50	0.18
	Cushion layer	27.04	6.50	0.15
	Soil matrix	29.04	8.50	10.00
Double-width unfilled roadbed	Concrete surface layer	5.00	4.50	0.24
	Foundation course	26.04	10.01	0.18
	Cushion layer	27.04	11.01	0.15
	Soil matrix	29.04	13.01	10.00
Three unfilled excavation roadbeds	Concrete surface layer	5.00	4.50	0.24
	Foundation course	26.04	14.52	0.18
	Cushion layer	27.04	15.52	0.15
	Soil matrix	29.04	17.52	10.00
Double low embankments	Concrete surface layer	5.00	4.50	0.24
	Foundation course	26.04	10.01	0.18
	Cushion layer	27.04	11.01	0.15
	Soil matrix	29.04	23.01	10.00
Double high embankment	Concrete surface layer	5.00	4.50	0.24
	Foundation course	26.04	10.01	0.18
	Cushion layer	27.04	11.01	0.15
	Soil matrix	29.04	87.01	20.00
Double-width cutting	Concrete surface layer	5.00	4.50	0.24
	Foundation course	26.04	10.01	0.18
	Cushion layer	27.04	11.01	0.15
	Soil matrix	29.04	27.01	10.00

**Table 3:** Material parameters of each structural layer.

Structure	Elastic Modulus (MPa)	Density (kg/m^3^)	Poisson Ratio	Damping Ratio
Concrete surface layer	30,000	2400	0.15	0.05
Foundation course	1500	2300	0.25	0.05
Cushion	500	2200	0.35	0.05
Earth base	30	1800	0.40	0.05

## Data Availability

The original contributions presented in this study are included in the article. Further inquiries can be directed to the corresponding author.
